# Viral Entry Properties Required for Fitness in Humans Are Lost through Rapid Genomic Change during Viral Isolation

**DOI:** 10.1128/mBio.00898-18

**Published:** 2018-07-03

**Authors:** Sho Iketani, Ryan C. Shean, Marion Ferren, Negar Makhsous, Dolly B. Aquino, Amedee des Georges, Bert Rima, Cyrille Mathieu, Matteo Porotto, Anne Moscona, Alexander L. Greninger

**Affiliations:** a Department of Microbiology and Immunology, Columbia University Medical Center, New York, New York, USA; b Department of Laboratory Medicine, University of Washington, Seattle, Washington, USA; c Vaccine and Infectious Diseases Division, Fred Hutchinson Cancer Research Center, Seattle, Washington, USA; d Department of Chemistry and Biochemistry, Advanced Science Research Center, City College of New York, New York, New York, USA; e Center for Experimental Medicine, Queens University, Belfast, Northern Ireland, United Kingdom; f Center for Host-Pathogen Interaction, Columbia University Medical Center, New York, New York, USA; g Department of Pediatrics, Columbia University Medical Center, New York, New York, USA; h Department of Physiology & Cellular Biophysics, Columbia University Medical Center, New York, New York, USA; Vanderbilt University Medical Center

**Keywords:** entry mechanisms, lung infection, metagenomics, parainfluenza virus, paramyxovirus, viral evolution, viral fitness, viral fusion

## Abstract

Human parainfluenza viruses cause a large burden of human respiratory illness. While much research relies upon viruses grown in cultured immortalized cells, human parainfluenza virus 3 (HPIV-3) evolves in culture. Cultured viruses differ in their properties compared to clinical strains. We present a genome-wide survey of HPIV-3 adaptations to culture using metagenomic next-generation sequencing of matched pairs of clinical samples and primary culture isolates (zero passage virus). Nonsynonymous changes arose during primary viral isolation, almost entirely in the genes encoding the two surface glycoproteins—the receptor binding protein hemagglutinin-neuraminidase (HN) or the fusion protein (F). We recovered genomes from 95 HPIV-3 primary culture isolates and 23 HPIV-3 strains directly from clinical samples. HN mutations arising during primary viral isolation resulted in substitutions at HN’s dimerization/F-interaction site, a site critical for activation of viral fusion. Alterations in HN dimer interface residues known to favor infection in culture occurred within 4 days (H552 and N556). A novel cluster of residues at a different face of the HN dimer interface emerged (P241 and R242) and imply a role in HPIV-3-mediated fusion. Functional characterization of these culture-associated HN mutations in a clinical isolate background revealed acquisition of the fusogenic phenotype associated with cultured HPIV-3; the HN-F complex showed enhanced fusion and decreased receptor-cleaving activity. These results utilize a method for identifying genome-wide changes associated with brief adaptation to culture to highlight the notion that even brief exposure to immortalized cells may affect key viral properties and underscore the balance of features of the HN-F complex required for fitness by circulating viruses.

## IMPORTANCE

Human parainfluenza virus 3 is an important cause of morbidity and mortality among infants, the immunocompromised, and the elderly. Using deep genomic sequencing of HPIV-3-positive clinical material and its subsequent viral isolate, we discover a number of known and novel coding mutations in the main HPIV-3 attachment protein HN during brief exposure to immortalized cells. These mutations significantly alter function of the fusion complex, increasing fusion promotion by HN as well as generally decreasing neuraminidase activity and increasing HN-receptor engagement. These results show that viruses may evolve rapidly in culture even during primary isolation of the virus and before the first passage and reveal features of fitness for humans that are obscured by rapid adaptation to laboratory conditions.

## INTRODUCTION

Human parainfluenza viruses are enveloped single-stranded negative-sense RNA viruses in the *Paramyxoviridae* family that account for a significant amount of human respiratory illnesses, especially in infants, children, and hematopoietic cell transplant patients and other immunocompromised individuals (1, 2). Of these, human parainfluenza virus 3 (HPIV-3) is the most commonly isolated parainfluenza virus (3). It has recently become clear that the process whereby HPIV-3 binds and enters host cells is finely tuned for interaction with the correct host cell environment (4–7).

Two surface glycoproteins of HPIV-3, the hemagglutinin-neuraminidase (HN) and fusion (F) proteins, work in concert to mediate fusion into the target host cell during infection. HN is a tetramer that carries out several functions in addition to serving as the receptor binding protein for the virus by binding to sialic acids (8). When HN is bound to a receptor, it activates F to a fusion ready state. Once activated, F inserts itself into the target membrane and undergoes a series of conformational changes that lead to fusion. Structural rearrangement of the F protein drives the cell-associated fusion peptide toward the viral membrane, bringing the two membranes into proximity and facilitating membrane fusion and viral entry (9, 10). HN also stabilizes the prefusion state of F prior to receptor engagement (11) and cleaves the receptor via its neuraminidase activity during viral budding (9, 12). These properties act together during infection of a target cell, when HN must signal the F protein, inducing the series of conformational shifts in F that result in fusion of the viral membrane with the host cell membrane at the correct time and place in the highly specialized human airway microenvironment (7). Such critical functions place the HN protein and HN-F fusion complex under significant selective pressure.

Each feature of HN, and the balance between the pro- and antifusion properties of the HN-F fusion complex, modulates fitness for infection of humans (6), meaning in this study, the relative ability to replicate in a given host tissue. Significant differences in properties governed by HN and the HN-F interaction exist between cultured laboratory and clinical strains in the HN and F proteins, and these differences point to the requirements for infection in each environment (4). HPIV-3 adapts to culture via increased fusion activity, whether by HN’s increased avidity for receptor, enhanced activation of F, or reduced receptor cleavage that results in enhanced receptor contact and fusion (4–7). Evaluation of a panel of clinical strains that were deep sequenced without passage in immortalized monolayer cultures and which were grown only in primary human airway epithelium (HAE)—revealed functional differences in the HN-F fusion complex and a phenotype of less active fusion activation in the clinical viruses (4). The preservation of these functional features in the panel of clinical isolates during growth in human airway suggested that mitigating fusion may be important for fitness *in vivo*.

Such stark differences in pressures exerted by *in vitro* and *in vivo* conditions have notable consequences which have been observed and characterized in other systems in addition to HPIV-3. For example, passaging of influenza A virus in eggs during vaccine production results in adaptations that alter its antigenicity (13, 14), and cell culture adaptation of hepatitis C virus impairs its fitness *in vivo* (15). These and other studies (16–22) have delineated the importance of characterizing clinical viruses, as well as the need to investigate viral evolution in authentic tissues and the biological significance of such adaptations. In fact, based on evaluation of a set of clinical strains compared to laboratory strains and to other available sequences (4), we hypothesized that adaptation to culture conditions might occur so rapidly that by virtue of growing the viruses to sequence, the strains published as being clinical may have actually altered to suit cultured cells and could no longer be considered clinical strains. There has been a relative dearth of sequence information available for HPIV compared to other respiratory viruses (23), and more importantly a lack of information about strains prior to passaging in the laboratory and thereby being subjected to selective pressure.

To address the question of which features associated with fitness in humans may be lost in routine viral culture, we sought to understand the genomic changes associated with brief exposure of HPIV-3 from clinical samples to culture during the isolation process. We used metagenomic next-generation sequencing (mNGS) of field strains of HPIV-3 isolated from humans to analyze the diversity and commonalities of circulating HPIV-3 strains at the genomic level, and we simultaneously sequenced paired specimens that were subjected to culture for viral isolation. The sequencing strategy we used to sequence clinical strains directly without passage in immortalized cells does not rely on designing sequence-specific primers that may amplify only a subset of samples (in case of mutations) (24). Deep genomic sequencing of nine sets of paired clinical samples (primary nasal swabs in viral transport medium) and culture isolates (culture harvest from zero passage virus) led to discovery of a number of HN mutations associated with rapid evolution in culture. To assess the frequency of mutations identified earlier, we also performed deep sequencing of 118 HPIV-3 clinical samples and culture isolates from the University of Washington Virology Laboratory, allowing us to confirm that the alterations associated with brief exposure to culture for viral isolation were almost entirely found in the sequences of culture isolates and found commonly within populations of viruses in those isolates.

Functional characterization of the effects of several of the specific HN gene mutations found in the cultured isolates in a clinical isolate (CI) background—in expressed HN-F complexes or recombinant viruses bearing the mutated genes—reveals that the culture-associated mutations all increase fusion promotion by the HN-F complex. These results support the notion that clinical strains differ from cultured viruses in the balance of fusion properties. The locations of the residues that differ after brief culture of clinical viruses point to specific domains that are relevant to the control of viral entry *in vivo*. The sequencing data, when correlated to functional data, help define the flexibility and constraints of the HPIV-3 fusion complex. These data provide the first characterization of culture-associated HN mutations in the background of HPIV-3 clinical strains directly from patient samples. We show that, even during transient exposure to culture (“zero passage” virus), the act of isolating a virus is associated with genomic evolution that may significantly affect viral protein function and not reflect *in vivo* dynamics.

## RESULTS

### Mutation and mixed allele frequency comparison between clinical samples and their matched cultured specimens.

To compare HPIV-3 sequenced directly from patients with HPIV-3 sequenced after brief growth in culture, nine HPIV-3-positive (HPIV-3+) nasal swabs were sequenced metagenomically, and their paired viral isolates were sequenced after initial isolation in rhesus monkey kidney (RhMK) cells. We investigated the variant alleles with a minor allele frequency of >5% that arose in culture. The nine swabs included one clinical sample that was independently cultured twice, such that ten viral isolates were sequenced. Clinical respiratory samples that tested positive for HPIV-3 by quantitative reverse transcription-PCR (qRT-PCR) with cycle thresholds under 25 were either sequenced directly by metagenomic next-generation sequencing (mNGS) without exposure to culture or grown on RhMK cells and viral isolates were harvested and sequenced. The choice of RhMK cells for this study was based on the fact that these are the standard cells used for viral isolation by many or most diagnostic microbiology laboratories and would allow us to answer the question of whether this standard process leads to alteration in the viruses.

Seven of the nine cultured isolates were harvested before day 5 of culture, and no viruses were passaged beyond initial isolation. Sequencing reads for the cultured viruses were analyzed for mutational changes relative to the HPIV-3 consensus genome obtained from metagenomic sequencing of the original clinical sample (without exposure to any cell culture), and both assemblies were analyzed for variants with >5% minor allele frequency (MAF) (25).

**FIG 1:**
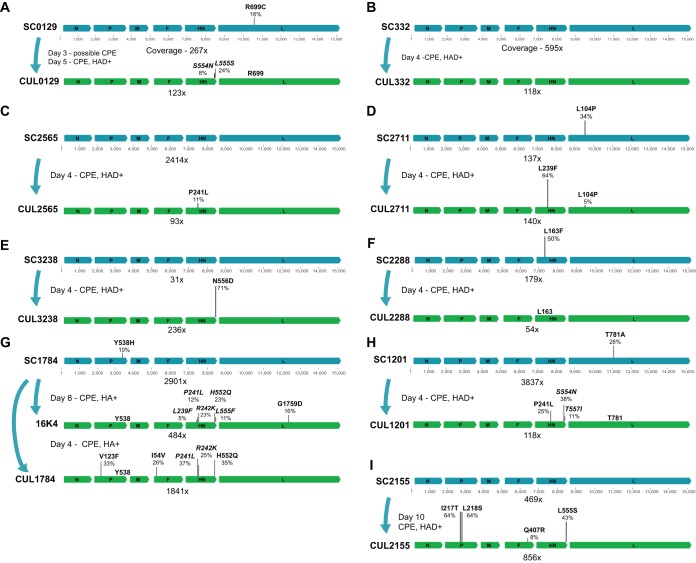
Mutational changes and allele frequencies in matched clinical samples and cultures. Blue genomes represent clinical samples, and the green genomes below the blue genomes represent the same sample after it was inoculated into culture and harvested several days later. The number of days cultured, hemagglutinin adsorption assay results (HAD), and observed cytopathic effect (CPE) are shown for each viral isolate. Virus from sample SC1784 in panel G was isolated twice. Coverage is averaged across the entire genome and depicted underneath each genome. Allele frequencies are plotted to scale above the genome organization. Mutational changes are described relative to the amino acid in the consensus genome of the original patient sample. Variants indicated in italics were found exclusively on separate reads and are considered unlinked.

A total of 28 nonsynonymous mutational changes with MAF change of >5% in response to exposure to cell culture were found across the nine sets of HPIV-3 viral isolates, with 18 of the 28 in the HN protein (*P* < 0.001 by Fisher’s exact test) (Fig. 1). Of the ten mutations found outside the HN protein, four were found in the P protein, four in the L protein, and two in the F protein. Of the two L variants that increased in allele frequency in cultured specimens, one (G1759D) was not found in a separate viral isolation performed on the same clinical sample, while the other (P104L) was found at a high allele frequency in the original clinical sample (Fig. 1D to G). Evolution of the polymerase complex will be explored separately, as the focus of this current paper is on the entry complex.

During viral isolation from sample SC1784, we noted the appearance of the previously described HN H552Q mutation at 35% MAF after only 4 days in culture (Fig. 1G). HN H552Q has previously been isolated in laboratory-adapted HPIV-3 strains and is associated with enhanced fusion promotion as a result of HN’s F-triggering function (7, 26–28). Two independent viral isolations in RhMK cells of this clinical sample resulted in selection for the HN H552Q variant allele.

During viral isolation in RhMK cells from sample SC3238, an HN N556D mutation emerged from 0% to 71% allele frequency in only 4 days, the greatest change in allele frequency during the brief exposure to culture (Fig. 1E). Previous comparison of direct clinical isolates with laboratory-adapted strains showed that every clinical strain bore asparagine (N) at position 556, while our laboratory strains bore aspartic acid (D) at position 556 (4). The HNs derived from clinical isolates (CIs) showed from 4- to 10-fold-higher neuraminidase activity than HNs derived from the lab-adapted strains, and the substitution of the aspartic acid at this position in the laboratory strains led to an approximately 5-fold-lower receptor cleavage in the laboratory-adapted strains, suggesting that the asparagine at position 556 is essential for the high propensity for receptor cleavage in field strains. The rapid emergence of this adaptation to culture by reducing receptor cleavage is remarkable.

During viral isolation from both samples SC0129 and SC2155, an HN L555S mutation, adjacent to residues 552 and 556 mentioned above, arose to 24% and 43% MAF (Fig. 1A to I). During isolation of sample SC1784, the same site contained an L555F mutation that was unlinked to the H552Q mutation that also arose during that isolation (i.e., was found exclusively on separate reads). The SC1201 isolation also resulted in selection of novel mutations HN S554N and HN T557I, comprising a cluster from positions 552 to 557 with significant alterations in the cultured viruses (Fig. 1H).

While the region of HN corresponding to the cluster at positions 552 to 557 above was known to be of interest for entry *in vivo* (based on features of H552 and N556), during independent viral isolations from sample SC1784, a set of mutations emerged in HN at residues previously unrecognized to be relevant to fitness: P241 and R242 (HN P241L and HN R242K). These mutations were not linked to each other. The P241L mutation described above was also recovered during viral isolations of SC2565 and SC1201 (Fig. 1C to H). Adjacent to these residues, another previously undescribed HN mutation L239F was selected at a high allele frequency (64%) during isolation of SC2711 and at a low level during isolation of SC1784 (5% MAF in 16K4 and 3% MAF in CUL1784). As discussed further below, these three residues describe a region of interest based on our previous structural analysis of the HN dimer interface (6).

### Phylogeny and HN mutation history in original samples.

**FIG 2:**
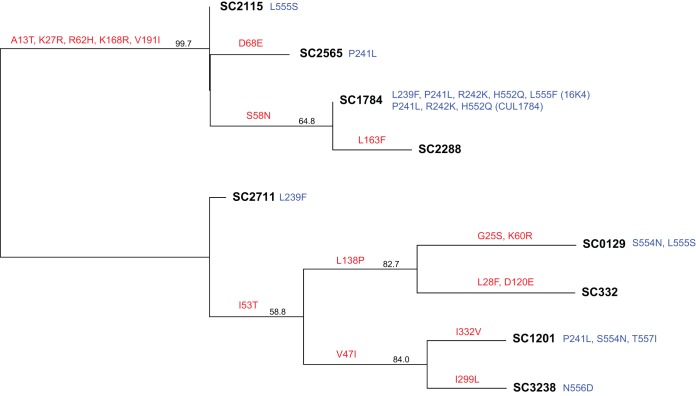
Neighbor-joining phylogenetic tree of the original patient sample HPIV-3 HN proteins used in the matched viral isolate sequencing experiment. Red mutations are the phylogeny-determining mutations found on each branch lineage. Blue mutations are the minor allele mutational changes found during isolation of each particular clinical sample. Two different sets of mutational changes are listed for sample SC1784. Consensus support values are shown next to branches.

To dissect the influence exerted by background HN sequence of the clinical virus on subsequent culture adaptation, we generated a phylogenetic tree based on amino acid substitution distance in the HN protein of our clinical samples (Fig. 2). None of the phylogeny-determining mutations among direct clinical sample strains were located in HN’s receptor binding site II or second dimer interface domain that emerged during rapid culture adaptation. Independent viral isolates from sample SC1784 (16K4 and CUL1784) produced P241L, R242K, and H552Q. The HN mutations L239F, P241L, S554N, and L555S also arose among separate lineages.

### Large-scale whole-genome sequencing of HPIV-3 direct- and culture-exposed strains shows that culture-associated mutations are rarely found in clinical strains.

To place the alleles that arose in the paired clinical-culture specimens above in the broader context of HPIV-3 sequence information, we used mNGS to obtain whole genomes from an additional 118 HPIV-3 strains, 95 of which were primary viral isolates (passage zero virus; brief exposure to culture only during viral isolation) and the remaining 23 were directly from patient samples. All specimens were collected in 2015 or 2016 (see Table S1 in the supplemental material). Recombination analysis using RDP4 (29, 30) revealed no recombination among these strains similarly to other nonsegmented negative-strand RNA viruses (29, 30). Phylodynamic analysis of the concatenated coding regions of the 118 HPIV-3 sequenced strains revealed a mean mutation rate of 5.948E−4 substitutions/site/year (95% confidence interval, 3.8463E−4 to 7.1847E−4). Analysis of concatenated coding regions from all 243 HPIV-3 sequences with complete coding sequences (CDS) in the GenBank NT database as of 7 September 2017 revealed a similar mean mutation rate of 3.12E−4 substitutions/site/year (95% confidence interval, 2.33E−4 to 4.02E−4) similar to those of other paramyxoviruses (31).

10.1128/mBio.00898-18.1 TABLE S1  Sample metadata, including GenBank accession numbers for consensus HPIV-3 genomes. Download TABLE S1, PDF file, 0.1 MB. Copyright © 2018 Iketani et al. 2018 Iketani et al. This content is distributed under the terms of the Creative Commons Attribution 4.0 International license.

**FIG 3:**
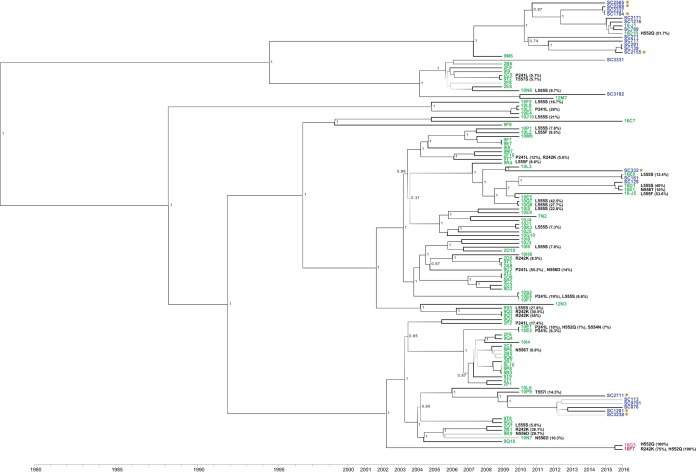
Whole-genome sequencing of 95 HPIV-3 primary viral isolates and 23 HPIV-3-positive clinical samples from excess samples at the University of Washington Virology Laboratory. All specimens were collected from 2009 to 2016. Clinical “SC” HPIV-3 genomes shown in blue were sequenced metagenomically directly from patient samples, while viral isolates are shown in green. HPIV-3 sequences from clinical samples from the original matched sample are shown as brown stars. At the bottom, 16F7 is shown in red, as it is a likely culture-adapted College of American Pathologists proficiency testing sample, and 16G3 is pink because it was isolated over a 14-day period at the same time 16F7 was being cultured and is a potential 16F7 contaminant. Posterior support values are shown next to branches with gray scale gradient shading from black at 0.7 to very light gray at 0. The scale at the bottom of the figure shows the collection date for each sample.

Phylogenetic analysis of the newly sequenced HPIV-3 coding sequences revealed multiple lineages of HPIV-3 present in 2015–2016 clinical samples and clustering by time of collection as the dominant feature rather than by sequence obtained from viral isolate versus clinical sample (Fig. 3). However, examination of the deep sequencing data for 36 of the 95 viral isolates revealed mutations with >5% MAF in residues 241 and 242 and residues 552 to 557 selected during the paired sample-isolate analysis above. While the original clinical samples for these isolates are not available, none of the clinical samples sequenced metagenomically contained variant residues at these loci. Of the 36 strains with variant residues, only 3 viral isolates and 2 laboratory-adapted strains contained variant residues with >50% MAF that would be expected to be reflected in consensus sequence available publicly.

In addition to the newly derived HPIV-3 genome sequences, we also examined all complete and partial consensus HPIV-3 HN sequences available in the GenBank NT database as of 18 August 2017—a total of 1,178 sequences—in a search for the culture-associated mutations isolated here. Of the culture-associated mutations in the HN protein described above, P241L (3), S554N (1), L555F (4), L555S (7), N556D (13), and T557I (2) appeared exclusively in sequences from cultured isolates (28, 32–37). None of the consensus sequences in NCBI with a P241L mutation had a mutation in the 552 to 557 residue range (consensus sequence, 552-HKSLNT-557). The H552Q mutation appeared previously in 15 sequences, with the vast majority of these (11/15) occurring in culture isolates; note that as above, even sequences listed as clinical may have experienced brief exposure to culture conditions. The L239F mutation has not been reported in any consensus HPIV-3 HN sequence in NCBI.

Three instances of the R242K alteration were found in NCBI GenBank (28, 38). One of these was recovered using RT-PCR directly from clinical specimens during an HPIV-3 outbreak in a children’s hospital cancer ward in Barcelona, Spain, in summer 2012 (38). The remaining two R242K mutations reported were from a likely culture-adapted College of American Pathologists proficiency testing sample (strain 16F7, GenBank accession no. KY674982) and a primary culture isolate from Japan (DDBJ accession no. AB623431) (28).

### HPIV-3 culture adaptations localize to the dimer interface of HN.

**FIG 4:**
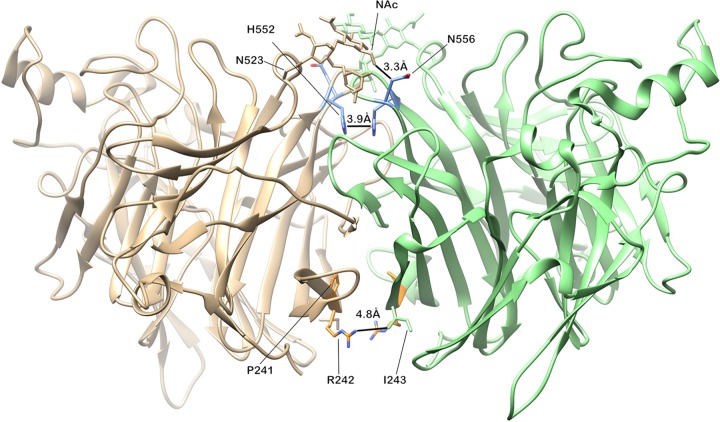
Mapping of minor alleles that arose during culture onto the PDB 4WEF HPIV-3 HN crystal structure. The diagram shows the interface between the globular heads of two monomers, including receptor binding site II known to be critical for activation of F (discussed in text). At the top are sites located between residues 552 and 556 where we found minor allele variations in culture. At the bottom of the diagram, residues 239 and 242 are shown, where minor allele variations also arose from culture. Side chains are shown as they are in the reference strain—not the mutations that we noted.

We mapped all the residue changes in the HN protein that arose during adaptation to brief culture onto the HPIV-3 HN protein crystal structure 4WEF (Fig. 4). All alterations localized to the dimer interface. We previously described critical residues at the HN dimer interface for HN’s roles in receptor engagement and activation of F at H552—which we identified as the secondary sialic acid binding site—and in receptor cleavage at N556, shown at the top of the HN globular head dimer interface in the diagram (Fig. 4). The diagram highlights the fact that N556 likely makes contact with the NAc modification of N523, and glycosylation could therefore have a significant effect on binding. We have shown that alterations that enhanced dimer contacts were associated with culture adaptation, while host fitness was correlated with reduced dimer contacts at these residues (6). Alterations in residues 552 and 556 have been associated with other culture conditions as well (5–7), supporting the notion that this site is intimately involved in the mechanism whereby HN activates F in the natural host. The mutations P241L and R242K have not been described before and appear in a cluster at the opposing side of the dimer interface. The rapid emergence of alterations here during adaptation of a patient’s HPIV-3 to culture suggests a role in viral entry in the natural host. Of note, P241 does not make specific contact with the opposing monomer, but a mutation would change the conformation of the loop significantly, thereby altering the contacts that the loop can have.

### HN mutations in site II and dimer interface modulate fusion promotion.

**FIG 5:**
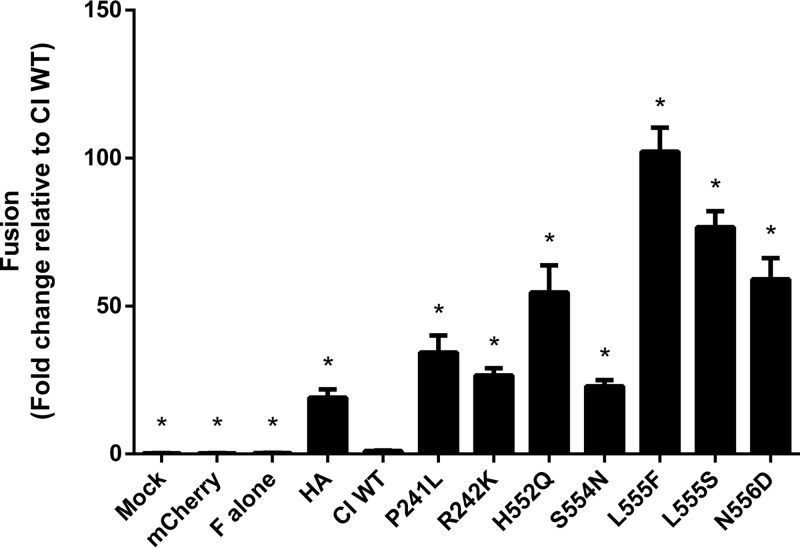
HN adaptations to culture increase fusion promotion by viral fusion complex. Cells were cotransfected with the indicated HN in a clinical isolate background, reference F, and the α fragment of β-galactosidase. These cells were then allowed to fuse with cells expressing the Ω fragment of β-galactosidase for 24 h. Fusion was stopped by lysing the cells, and β-galactosidase activity was quantitated and normalized to HN expression. Every mutation in HN conferred increased fusion promotion relative to the clinical wild-type HN. Results depict means plus standard errors of the means (SEM) (error bars) from three biological replicates consisting of three technical replicates each. Mutants were compared with CI WT with one-way analysis of variance (ANOVA) following log transformation of data. Values that are statistically significantly different (*P* < 0.0001) from the value for the CI WT are indicated by an asterisk.

To understand the selection forces that affect HPIV-3 fitness, we next analyzed the biological properties conferred by the culture-associated mutations in HN. Here we characterized the influence of each specific residue on the function of the HN-F fusion complex. To investigate whether the identified mutations altered HN’s fusion promotion ability we had previously observed in clinical isolates of HPIV-3, we introduced seven of these rapidly emerging mutations into the background of a well-characterized clinical isolate HN, clinical isolate 1 (CI-1) (4, 5). We paired these mutated HN proteins with a lab-adapted F to ensure that we could observe adequate levels of fusion to compare function, since clinical isolate HN and F pairs result in nearly unobservable levels of fusion (4). Fusion promotion by each of these mutant HN-F pairs was quantified with a β-galactosidase complementation assay and compared to the wild-type clinical isolate HN (CI WT) paired with the lab-adapted F (Fig. 5). We included an influenza A virus hemagglutinin (HA) and F pair as a positive control for fusion, as it had been previously shown that HN has a stabilizing property that prevents F triggering; that is, an increase in fusion relative to the CI WT would be expected and was seen (11). The fusion exhibited by all mutant HN-F pairs was considerably higher than that of the CI WT HN-F pair, ranging from a 20-fold increase (HN S554N) to a 100-fold increase (HN L555F).

### Neuraminidase activity is reduced by HN’s adaptations to culture except for H552Q.

**FIG 6:**
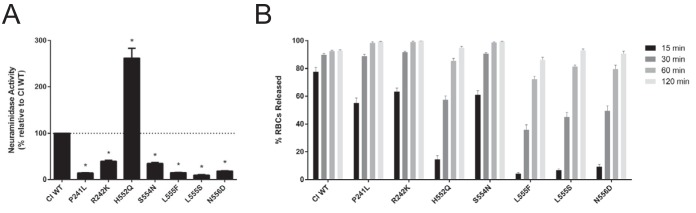
Increase in fusion promotion by mutant HN correlates with a decrease in neuraminidase activity and a decrease in RBC release. (A) Neuraminidase activity was quantified by measuring cleavage of 4-MUNANA. The observed values were normalized to expression of the HNs. Excluding H552Q (see Discussion), neuraminidase activity is decreased in all the mutant HNs compared to the wild-type clinical isolate. Results depict means plus standard deviations from three biological replicates consisting of three technical replicates each. Mutants were compared with CI wt with one-way ANOVA following log transformation of data. *, *P* < 0.0001. (B) Release of sialic acid-bearing RBCs by HNs at pH 7.5 and 37°C. 293T cells transiently expressing HNs were allowed to bind RBCs at 4°C for 30 min, washed, and transferred to 37°C. The percentages of RBCs released at 15, 30, 60, and 120 min were determined (*y* axis). All the mutants have decreased release of RBCs relative to the wild-type clinical isolate. Results depict means plus standard errors of the means (SEM) from three biological replicates consisting of two technical replicates each.

After observing that fusion promotion was increased by all HN mutants, we examined several interacting properties of HN (see introduction) that are involved in this process. We assessed the neuraminidase (receptor cleavage) activity and also quantified the release of receptor-bearing erythrocytes (RBCs) from HN-expressing cells as a measure of the balance between avidity and receptor-cleaving properties. Neuraminidase activity was measured through the cleavage of 2′-(4-methylumbelliferyl)-α-d-*N*-acetylneuraminic acid, sodium salt hydrate (4-MUNANA) by HN (Fig. 6A). As previously described, almost all the culture-adapted HN mutants displayed lower neuraminidase activity than CI WT, ranging from roughly 10% (L555S) to 40% (R242K) relative activity (4). The H552Q mutant had increased neuraminidase activity, with roughly 260% activity relative to CI WT. We have previously shown that this mutation at H552 on HN confers both higher receptor avidity and intrinsically enhanced triggering of F and thereby augments fusogenicity, an effect that overrides the increase in receptor cleavage (see the release assay below) (26).

Release of bound receptor-bearing RBCs by cell surface HNs reflects the balance of neuraminidase activity, which would release the bonds tethering the cells, and avidity of the HNs, which retain the bound cells (Fig. 6B). We quantified the amount of RBCs released at several time points when incubated at pH 7.5 and 37°C. Differences between the HNs was most readily observed at the earliest time point of 15 min, when CI WT had the most release (~77%), whereas the mutant HNs all had less release, reflecting either their higher receptor avidity, lower neuraminidase activity, or both. We observed two separate groups of mutants in their capability to release RBCs. A more rapidly releasing group—P241L, R242K, and S554N—had roughly 60% release at 15 min and complete release (~100%) after 120 min. In contrast, the more slowly releasing group, consisting of H552Q, L555F, L555S, and N556D, had 5 to 15% release at 15 min and 85 to 95% release after 120 min. As noted above, the higher receptor avidity of HN can override the higher-than-WT neuraminidase activity.

### Fitness of engineered recombinant HPIV-3 viruses is modulated by HN mutations.

**FIG 7:**
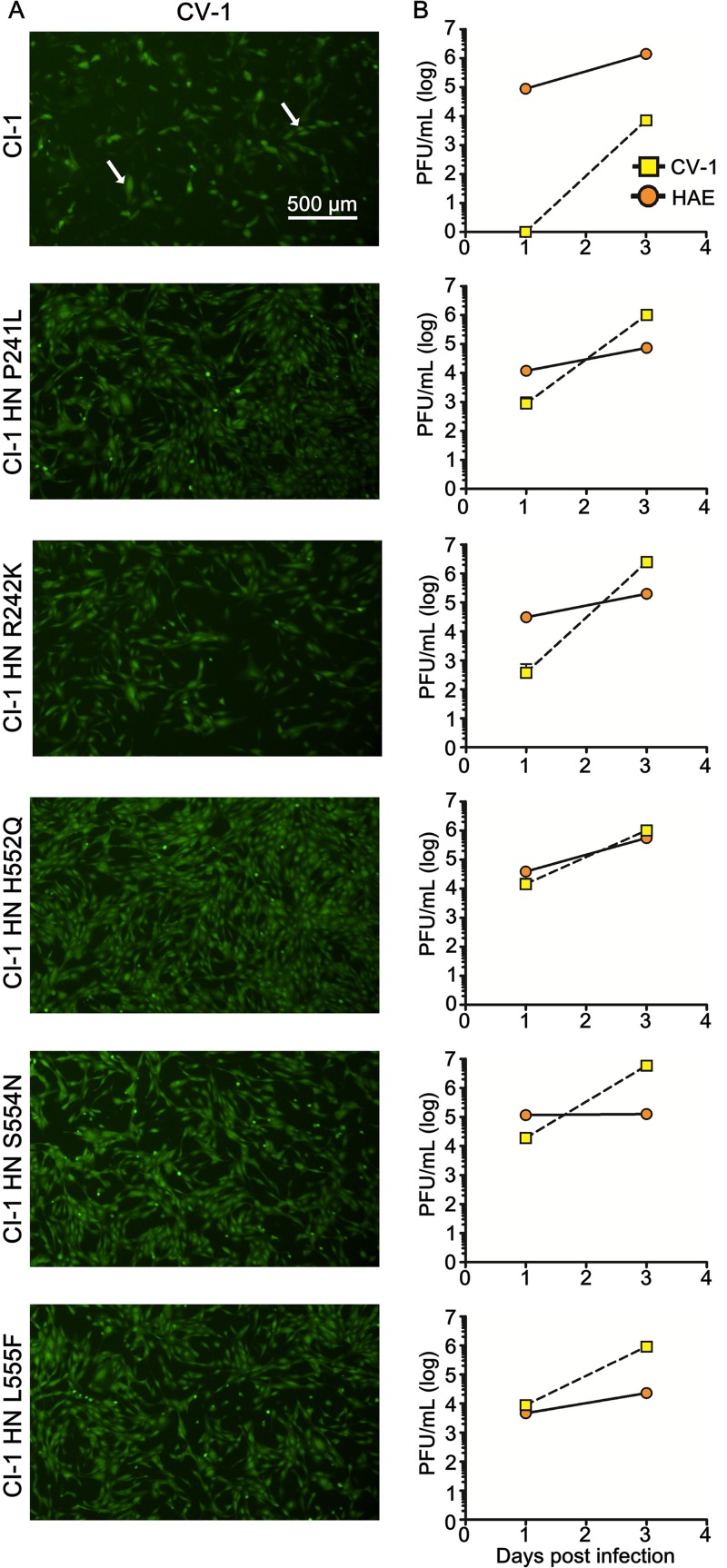
Growth of recombinant HPIV-3 CI-1 EGFP and recombinant HPIV-3 CI-1 EGFP bearing mutated HNs in CV-1 and HAE cells. (A) CV-1 cells were infected with 5,000 PFU of the different recombinant viruses (wt CI HN or panel of mutants). Infected cells were observed using an epifluorescence Nikon TS2R-FL inverted microscope 3 days postinfection. (B) Titration of HPIV-3 CI-1 EGFP viruses (wt CI or HN mutants) of either supernatant fluid from CV-1 cells (squares) or virus collected from HAE cells (circles) 1, 3, and 7 days after infection (PFU/ml). The values represent titers performed in triplicate from a representative experiment that has been repeated three times.

To assess the relevance of the HN adaptations in the context of authentic virus and determine the fitness of the culture-adapted viruses in immortalized cells and human airway epithelium (HAE), we generated recombinant viruses bearing the HN mutations of interest on the genetic background of our well-characterized HPIV-3 clinical virus CI-1 (4) and containing an enhanced green fluorescent protein (EGFP) cassette between the P and M genes (rHPIV3 CI-1–EGFP). Each engineered virus was sequenced in full to confirm the mutations of interest. These engineered viruses permit us to evaluate the outcome of selection in terms of impact on fitness. HAE cultures at an air-liquid interface or CV-1 immortalized cell monolayer cultures were infected with viruses bearing wild-type (wt) CI HN, CI HN P241L, CI HN R242K, CI HN H552Q, CI HN S554N, and CI HN L555F and expressing EGFP. Compared to the wt CI, the viruses bearing all the mutated HNs show more infected cells in the CV-1 cell monolayers after 3 days (Fig. 7A, arrows point to examples of individual infected cells); contrast wt CI-1 infection in the top photo to infection with viruses bearing individual HN mutations in the subsequent photos, where these adaptations confer superior growth in cultured cells as expected. The wt CI virus is handicapped in growth at day 1 compared to each of the viruses bearing the individual mutations in HN (Fig. 7B, yellow squares), producing no detected progeny compared to a titer of 10^3^ to 10^4^ PFU/ml for each of the viruses bearing a single adaptive HN mutation. The growth in CV-1 cells reveals that while the clinical virus is severely handicapped in monolayer culture, the mutations that emerged during the RhMK culture isolation process confer growth in these monolayer cultures. While the wt CI shows negligible fitness in monolayer culture compared to the adapted viruses on day 1, by day 3 infectious virus is released into the supernatant fluid of the virus to cell culture. At day 7 however, adaptive mutations have appeared (Table 1) and correlate with increased production of infectious particles (Fig. 7B, yellow squares).

**TABLE 1:** Allele frequency of CI-1 HN and mutant HPIV-3 strains

Strain	HN residue	Allele frequency (%)^a^		
		P0	CV1 7 days	HAE 7 days
CI-1	D216	97	90	100
	E216	3	10	0
	K553	100	95	99
	E553	0	5	0
	L555	100	88	100
	F555	0	12	0

CI-1 HN P241L	L241	99	99	100
CI-1 HN R242K	K242	100	99	99
CI-1 HN H552Q	Q552	100	99	100
CI-1 HN S554N	N554	99	99	99
CI-1 HN L555F	F555	99	100	100

a Allele frequency at passage zero (P0) or after growth on CI-1 virus (CV1) for 7 days or on human airway epithelium (HAE) for 7 days.

In HAE tissue, the wt CI-1 has a growth advantage over all the mutated viruses, showing that the individual culture-adaptive HN mutations modestly impair infection of HAE (Fig. 7B, orange circles). At day 7 after HAE infection, wt CI releases 1.8 × 10^5^ infectious particles into the HAE apical surface, which is between 3 and 80 times more particles than released by the mutant viruses. As expected based on our previous studies (4), growth in HAE did not result in emergence of mutations (Table 1).

## DISCUSSION

Mechanisms that affect respiratory virus-cell interplay in the context of humans are finely tuned to the environment of the host (4, 5). To understand natural viral infection and the features that govern infectivity and transmissibility in humans, we have endeavored to analyze field strains in airway tissues. We have shown for human parainfluenza virus type 3 that conclusions drawn from laboratory strains can be misleading with regard to the receptor interaction and viral fusion properties that govern entry and fitness *in vivo* (4, 5). Analysis of clinical strains suggests that the HN-F fusion pairs of circulating HPIV-3 viruses maintain a balance of properties that result in an inverse correlation between fusion in cultured cells and growth *in vivo*; the fusion complex operates under specific constraints that govern viability in the different environments of cell cultures and human beings.

We have previously used whole-genome sequencing of field strains of HPIV-3 isolated from humans and grown only in airway cultures to analyze the diversity and the commonalities of circulating strains of HPIV-3 (4). The sequences were used to define functional and structural properties of proteins of circulating strains in order to identify the genetic basis for properties that confer fitness in the field. While laboratory-adapted and CI airway-grown strains shared the basic properties of the HN-F fusion complex—receptor binding, fusion triggering, receptor cleavage—the balance between these properties at each step is shared across all CIs we studied and different from all laboratory strains. The CI fusion pairs all possessed lower fusogenicity and decreased F activation compared to those of laboratory strains.

Here we carried out deep genomic screens of paired HPIV-3 clinical samples; one portion of each sample was sequenced directly from the patient with no culture step, while the other portion was exposed to brief culture for viral isolation in immortalized rhesus monkey kidney (RhMK) cells, a standard cell line used in clinical laboratories for virus identification. We hypothesized that even during such brief periods of culture, mutations arising in the HN-F fusion complex could be informative about features essential for growth in humans that would be stringently selected against in culture. HPIV-3 evolution upon exposure to culture was extraordinarily rapid and almost entirely limited to the HN protein. Bioinformatic analyses of existing HN sequences revealed that HN mutations that arose during brief exposure to culture are almost uniformly found in consensus sequences from HPIV-3 that are either adapted to culture or exposed to culture—and not found in HPIV-3 sequenced directly from clinical samples. Deep sequencing of 118 newly derived HPIV-3 isolates and clinical samples revealed that more than one-third of HPIV-3 isolates had subpopulations of HN dimer interface variants that were not reflected in consensus sequences and were not present in HPIV-3 sequences obtained directly from clinical samples. The sequences were used to define functional and structural properties that differ from laboratory-selected circulating strains in order to identify the genetic basis for properties that may confer fitness in the field.

The HN mutations that we identified in response to exposure to culture included both novel and previously observed and characterized adaptations to immortalized cells. Remarkably, all the mutations map to the dimer interface and receptor binding site II of HN. These results point to the critical role of this region in the HPIV-3 entry mechanism and for fitness *in vivo*, as we have deduced from smaller-scale and mechanistic studies (4–7, 10). The H552 residue at receptor binding site II at the dimer interface of HPIV-3 HN influences HN’s dimerization and its activation of F (6, 7). We have shown that the presence of the glutamine at position 552 at the dimer interface of the HPIV-3 HN increases HN oligomerization and thereby modulates F triggering, with this HN more actively triggering F (6, 10). When the virus bearing this mutated HN was subjected to growth in human airway, it rapidly acquired a compensatory mutation, Q559R, which reduced fusion triggering and reduced HN dimerization. Structural analysis of footprints of the dimer interfaces showed that when HN H552Q is present in receptor binding site II, more of the interface residues are in contact with the opposing monomer face. However, in the HN with the Q559R compensatory mutation that permits growth in the airway, the dimer interface footprint shows decreased points of contact. The dimer interface footprint pointed to several residues and domains whose contact with the opposing monomer may be critical for the key functions governed by HN’s receptor binding site II. The emergence of the H552Q mutation after brief culture highlights both the flexibility of HN’s site II to adapt to new conditions and also the exquisite constraint of the virus’s fusion complex with respect to alterations in the environment.

Adjacent to the H552 residue in the dimer interface, but not making contact in the dimer interface footprint mentioned above, is N556, a residue we have previously shown to be highly conserved in all clinical strains. This residue was previously found to be an aspartic acid in laboratory-adapted strains, associated with a dramatic decrease in neuraminidase activity consistent with the overall high-receptor-contact/high-fusion laboratory-adapted phenotype. In the present study, the N556D mutation emerged during the very brief exposure to culture, highlighting the advantage of lowering neuraminidase activity to reduce receptor cleavage for culture conditions versus the importance of neuraminidase for human infection. Within the same cluster, S554 was altered during culture adaptation; this is a residue that contacts the opposing monomer in the dimer interface cluster (6, 39). The precise role of this residue in the functions of the dimer region will be explored.

Strikingly, several mutations emerged in residues at the opposite side of the HN dimer interface from the cluster at positions 552 to 556: residues 239, 241, and 242. The points of contact of N240 and R242 with the opposing HN monomer in the dimer interface footprint had been noted to correlate with fusion promotion and airway adaptation; however, the function of these residues had been unexplored (6). The selection for L239F, P241L, and R242K in the absence of mutations in the domain from positions 552 to 556 suggests that the newly identified important region in the HN dimer interface may act independently of receptor binding site II, and the functional relationships between these two sites will be explored in future studies. Numerous deep sequenced viral isolates also demonstrated P241L and R242K HN variants in the absence of mutations in the domain from positions 552 to 556. The finding that this second dimer interface domain undergoes rapid selection in the background of authentic clinical strains suggests that it may be relevant to fitness in the airway.

To investigate the biological significance of these adaptations, we generated singly mutated HN molecules in the background of our well-characterized HPIV-3 clinical virus HN, CI-1 (5), and generated recombinant viruses bearing the HN mutations on the genetic background of CI-1. Brief exposure to culture, through this diversity of strategies, resulted in increased fusion promotion for all the HN variants tested: H552Q, S554N, L555F, L555S, N556D, P241L, and R242K. While perhaps not unexpected based on our previous hypotheses, the absolute consistency of this finding—that through a range of specific mutations and specific functional and structural differences, the virus arrives at increased fusion—is remarkable. Each individual HN mutation conferred fitness for recombinant virus infection of immortalized monolayer culture cells and for spread of virus through the culture, compared to the parent wt CI.

To explore the components of HN’s properties that contribute to the enhanced fusion promotion and infection properties for all the emerging HN mutants, we assessed neuraminidase activity and HN’s release of receptor-bearing target cells, a measure of the balance between receptor avidity and cleavage (26). We have shown that clinical viruses, compared to laboratory strains, have a combination of high neuraminidase, low avidity, low F triggering, resulting in lower fusogenicity. The HN mutants that emerged here all except for HN H552Q had decreased neuraminidase activity. It is of interest that mutations at site II affect neuraminidase enzymatic activity, since this site is not known to possess enzymatic activity, which resides in the bifunctional active site at T193/D216 (12, 40–42). One mechanistic conjecture that will be explored is that alterations or interactions at one HPIV-3 HN site (site I or II) modulate the function of the other site, as we have shown for another paramyxovirus, Newcastle disease virus, where receptor interaction at HN site I leads to the activation of site II (43, 44). These data may suggest an unexplored cross talk between site I and site II that could alter neuraminidase activity allosterically. All the mutants—including HN H552Q—showed reduced release of RBCs compared to the clinical strain. The HN H552Q mutant’s decreased release of RBCs in the face of increased neuraminidase activity relative to the CI WT is in agreement with our previous finding that this mutation confers increased receptor avidity (26) that balances the receptor-cleaving activity for an overall retention of contact. Interestingly, the H552Q mutation in the lab-adapted HN background does not have higher neuraminidase activity (7), supporting the notion that the phenotype conferred by these sites may be modified in the context of the genetic background. Collectively, the pattern is of enhanced receptor engagement and an increase in fusogenicity that confers fitness *in vitro*. The genetically engineered viruses bearing the individual alterations in HN show that each is fit for growth in monolayer culture compared to the wt parent and less fit for growth in HAE. The mutations emerging during the brief viral isolation process thus led directly to fitness for growth in monolayer culture.

Interestingly, there is no single mutation or minor allele required for successful growth in culture, although all samples in this study did produce at least one nonsynonymous minor allele on either the F or HN proteins. The minor alleles were extremely biased toward mutations in the HN or F protein, with eight of ten cultures presenting minor alleles on the HN protein and four presenting a minor allele on the F protein, supporting the critical nature of the HN-F fusion complex for host adaptation (4). Intriguingly, single mutations in HN did not confer fatal growth disadvantage in the airway model, despite conferring fitness in monolayer culture. It will be of interest to determine whether an accumulation of several fusion-skewing mutations is necessary for an absolute inability to grow in airway and whether a more sensitive experimental system (i.e., infection *in vivo*) would be responsive to single alterations or only to a greater dimer interface disturbance.

The genome sequences presented here provide a significant resource for future HPIV-3 genomic epidemiology and biochemical characterization, increasing the number of publicly available genomes by more than 40% (45, 46). We obtained full genome sequence coverage from as few as an estimated 20,000 starting copies (4) and found that relatively uniform whole viral genome coverage can be achieved with 100,000 paired-end reads, suggesting that many samples could be sequenced in a single MiSeq run. Whole-genome viral sequencing from clinical samples should be both technically and economically feasible, permitting investigators to avoid the use of laboratory-adapted strains of virus in immortalized cultured cell lines. The deep genome-wide screens of paired clinical samples and viral isolates allow for simple, rapid interrogation of viral evolution in the context of multiple clinical strain backgrounds. The methods pursued here allowed for discovery of novel HN residues of interest that heretofore had not been identified based on consensus sequence. Deep sequencing after rapid selection pressure may allow for discovery of a greater number of novel mutations that may be removed from the population by drift or competition with other variant subpopulations after longer selection periods yet provide clues to function. Other techniques that can more comprehensively profile single mutations, such as deep mutational scanning, are often limited by their use of singular backgrounds, often in the context of culture-adapted strains, and focus on singular proteins.

Our results are consistent with an “uncertainty principle” for virology: the act of attempting to study a clinical viral sample through commonly performed laboratory procedures, such as viral culture in cell lines, affects the authenticity of the viral biology even before the first passage. That viruses evolve uniquely in culture has been shown for HPIV-3 (27); however, the extraordinarily rapid nature of evolution in culture, even without passage, is notable. Most of the adaptive genotypes could be found as minor alleles only by deep sequencing of clinical samples and viral isolates. With the increased provision of deep coverage viral genomes obtained directly from clinical samples due to diagnostic mNGS, experiments similar to the one outlined here will be critical for potentially pathogenic viruses in order to assess whether previous viral characterization studies apply to newly sequenced clinical samples (47, 48). For HPIV-3, our results are consistent with the notion that use of authentic culture models that better reflect HPIV-3 growth *in vivo* is critical if we are to understand and characterize pathogenesis (4).

The specific mutations that emerged in the cultured samples, while disturbing from the perspective of the “uncertainty” described above, provide a wealth of information about HN domains that may be critical for fitness in humans. In our focus on the correlation of emerging HN mutations to function, we did not characterize all combinations of HN mutations, and for functional studies, we paired HNs with a lab-adapted more fusogenic F protein in order to readily discern differences in HN properties; ongoing studies will explore the potential for epistatic interactions in HPIV-3 evolution. Of particular interest is the cluster of residues around P241 and R242 that were uncovered as critical in this study. The interactions of these residues with the opposing monomer, and these points of contact of this domain within the interface, were suggested based on structural data to be relevant for fusion promotion (6). Future studies will address the function of this HN dimer interface domain in HPIV-3 fusion and cell entry.

## MATERIALS AND METHODS

### Sample description.

The term “clinical sample” is used to describe primary nasal swabs in viral transport media, while “viral isolate” or “culture isolate” is used to describe culture harvest from zero passage virus (i.e., brief exposure to culture only during viral isolation). For paired clinical sample/culture isolate sequencing, 200 µl of nasal swab viral transport medium from nine patients from 2015 to 2016 with an HPIV-3 cycle threshold (*C*_*T*_) of <25 was inoculated onto primary rhesus monkey kidney (RhMK) cells (Quidel Diagnostics) and incubated on a roller drum at 37°C. The tubes were assessed for cytopathic effect daily and confirmed via hemadsorption with 1% guinea pig red blood cells (RBCs). Positive cultures were harvested and stored at −80°C.

Deidentified bronchial alveolar lavage (BAL) fluid and nasal swab samples from 23 patients from 2016 used for prior clinical testing were stored at −80°C and subjected to mNGS. In addition, 95 culture harvests for which viral isolation was performed for prior clinical testing of BAL fluid samples, nasal swabs, and sputum samples from 2009 to 2016 at the University of Washington Virology Laboratory were also stored at −80°C and subjected to mNGS. Culture harvests from 2009 to 2016 were performed using the same protocol detailed above for the 2016 paired sequencing experiment. Use of excess deidentified clinical specimens was approved by the local University of Washington institutional review board (IRB) (Protocol STUDY00000408). Sample metadata, including GenBank accession numbers for consensus HPIV-3 genomes, are available in Table S1 in the supplemental material and associated with NCBI BioProject 338014.

### mNGS and amplicon sequencing.

mNGS was performed as previously described (49). RNA was extracted from 100 µl of thawed culture harvests and clinical samples using the Zymo Viral RNA kit (Zymo Research) and treated with Turbo DNase I (Thermo Fisher) for 30 min at 37°C (50). Double-stranded cDNA was made using random hexamers, SuperScript III reverse transcriptase (Thermo Fisher), and Sequenase v2.0 (Thermo Fisher) and cleaned using Zymo-5 DNA Clean and Concentrator (Zymo Research). mNGS libraries were produced by adding one-third volume of NexteraXT (Illumina) and finished with 19 cycles of dual-indexed PCR amplification and cleaned using 0.8× Ampure beads (Beckman Coulter) (51). Libraries were sequenced using one 192-bp and two 300-bp runs on an Illumina MiSeq system. Libraries from the matched viral isolates were prepared twice in independent library preparations and sequencing runs to control for artifacts of library preparation.

Amplicon sequencing was performed to investigate linkage between mutations recovered during mNGS. Reverse transcription-PCR (RT-PCR) was performed using 35 cycles of the default parameters (melting temperature [*T*_*m*_], 55°C) in the One-Step RT-PCR kit (Qiagen) with PCR primers HPIV3-7477F (F stands for forward) (5′-TACAGATAGGGATAATAACTGTAAA-3′) and HPIV3-8635R (R stands for reverse) (5′-GCTTTGCTCCTAAGTTTTTTATATT-3′). Amplicons were purified using 1.0× Ampure beads, and dual-indexed TruSeq libraries were prepared using the Kapa HyperPrep kit with eight cycles of PCR amplification (52).

### Sequence data alignment and analysis.

Sequencing reads were adapter and quality trimmed using cutadapt and then aligned to the HPIV-3 reference sequence (NC_001796) with Geneious v9.1.7. Consensus genomes were extracted for original patient samples, and culture sample sequencing reads were aligned to the sample consensus. Consensus genomes were visualized and mutations and minor alleles with coverage of >50× and minor allele frequency (MAF) of >5% were called using Geneious 9.1.7. The known locus of RNA editing in the phosphoprotein gene was not included among the variant nonsynonymous alleles, nor were adenosine deaminase-like changes present on the same strand in P0 virus (affecting minor variants at R192G and I210V in CI-1 in Table 1).

### Phylodynamic analysis.

For phylodynamic analysis, coding nucleotide sequences from the 118 newly generated HPIV-3 genomes sequenced were extracted, concatenated, and aligned using Genious v9.1.7 and MAFFT (53). The resulting alignment was used to generate a Bayesian Skyline tree with an uncorrelated relaxed clock and Hasegawa, Kishino, and Yano (HKY) substitution model with gamma plus invariant site heterogeneity model (four gamma categories) and three codon partitioning in BEAST v1.8.4 with a Markov chain Monte Carlo (MCMC) length of 10,000,000 iterations (54). Tree generation trace information and evolutionary clock rates were gathered and analyzed using Tracer v1.6.0 (55). The resulting trees were annotated and compiled using TreeAnnotator v1.8.4 and then annotated and scaled in FigTree v1.4.2. The same phylodynamic protocol was also performed on all complete HPIV-3 genomes present in NCBI NT as of 17 August 2017. Recombination detection was performed using RDP4’s RDP, Chimaera, MaxChi, GENECOV, SiScan, and 3Seq algorithms with default settings (29).

To determine whether culture-associated HN amino acid changes were present in our paired sample sequencing, we downloaded all partial and complete HPIV-3 HN sequences from NCBI NT as of 17 August 2017. Metadata on whether specific sequences originated from clinical sample or culture material were gathered from the NCBI GenBank sequence record and associated manuscripts in PubMed. However, it is not always possible to determine whether there was brief exposure to culture conditions prior to sequencing. To show how the background of HN protein sequence could influence the amino acid mutations selected during culture, a majority consensus amino acid sequence of HN protein from each putative clinical sample was extracted and aligned using MAFFT. A neighbor-joining tree was created using the Genious tree building algorithm, Jukes-Cantor distance method, and 100,000 replicates.

### Functional characterization of HPIV-3 HN.

The HN mutants examined in this study were generated through site-directed mutagenesis of the previously constructed pCAGGS mammalian expression vector with the CI-1 HN gene (4). Plasmids were Sanger sequence confirmed prior to usage. Transfections with plasmids were performed in HEK 293T cells using Lipofectamine 2000 in accordance with the manufacturer’s instructions. Cell surface expression of each HN was measured through standard immunofluorescence assay procedures using monoclonal antibodies against HPIV-3 HN and used for normalization as appropriate. Two monoclonal anti-HN antibodies used here (VT-3G9-B9 and VT-10F7-B11) were custom-made by Aldevron using DNA immunization. Briefly, a cDNA encoding HPIV-3 HN was cloned into expression plasmids (pEGFP) (56). Groups of laboratory rats (Wistar) were immunized by intradermal application of DNA-coated gold particles using a handheld device for particle bombardment (gene gun). Serum samples were collected after a series of immunizations and tested in flow cytometry on HEK cells transiently transfected with the above expression plasmids. Antibody-producing cells were isolated and fused with mouse myeloma cells (Ag8) according to standard procedures.

### Measurement of fusion promotion.

The ability of individual HNs to promote fusion was measured through a β-galactosidase complementation assay as previously described (26, 40). HNs were paired with the F protein from a lab-adapted strain (5), transiently transfected in HEK 293T cells, and then cell fusion was quantified. Lab-adapted F partner molecules were used as previously described (4, 5), since fusion is minimal when CI HN and F are expressed in pairs, and does not allow for quantitative comparisons.

### Measurement of neuraminidase activity.

Neuraminidase activity of HNs were measured as previously described (7, 41). HEK 293T cells were transiently transfected with individual HNs, collected through detachment with 5 mM EDTA, washed with Dulbecco phosphate-buffered saline (DPBS) supplemented with 1 g/liter d-glucose and 36 mg/liter sodium pyruvate (Gibco), then resuspended in CO_2_-independent medium (pH 4.7) (Gibco). Cells were then added to 2′-(4-methylumbelliferyl)-α-d-*N*-acetylneuraminic acid, sodium salt hydrate (4-MUNANA) at a 1:1 ratio for a final concentration of 10.2 mM. Fluorescence was measured every 2 min for 1 h at 37°C with a microplate reader (Tecan).

### Measurement of RBC release.

The ability of HNs to release bound RBCs was measured as described previously (9, 26). RBCs were bound to cells for 30 min at 4°C and washed to remove unbound RBCs. CO_2_-independent medium (pH 7.5) was then added to the cells, and the cells were incubated at 37°C for the appropriate times before being collected and replaced for the next time point. After 120 min, RBCs that remained bound to the cells were lysed with ACK (ammonium-chloride-potassium) lysing buffer (Gibco) and quantified by measurement of absorbance at 410 nm.

### Immortalized cell culture.

CV-1 (African green monkey kidney) and 293T (human kidney epithelial) cells were grown in Dulbecco’s modified Eagle’s medium (DMEM) (Cellgro; Mediatech) supplemented with 10% fetal bovine serum (FBS) and antibiotics at 37°C in 5% CO_2_.

### Recombinant virus production and analysis.

rHPIV3 clinical isolate 1 (CI-1)-EGFP is a recombinant virus generated with a plasmid coding for the majority consensus sequence of HPIV-3 CI-1 (4, 5, 7) obtained by deep sequencing and containing an enhanced green fluorescent protein (EGFP) expression cassette between P and M viral genes. All mutants were derived from this unique sequence. Recombinant viruses were generated by reverse genetics in the rHPIV3-CI-1-EGFP-F K108 background using a modification of methods previously described (57). Briefly, 293T cells were seeded in six-well plates. The following day, the cells were transfected using ProFection mammalian transfection system, calcium phosphate kit (Promega) according to the manufacturer’s recommendations with 10 µg of pFLC plasmid encoding the full-length viral genome, 40 ng of pCAGGS-HPIV3 P, 40 ng of pCAGGS-HPIV3 L, 200 ng of pCAGGS-HPIV3 N, 200 ng of pCAGGS-T7 polymerase, 100 ng of pCAGGS-HPIV3 lab-adapted HN, and 100 ng of pCAGGS-HPIV3 lab-adapted F (4). The following day, supernatant fluids were replaced with DMEM supplemented with 10% FBS. Two days after transfection, the cells were mechanically detached by pipetting up and down and overlaid onto CV-1 cells in a 10-cm culture dish. Cultured cells were monitored by fluorescence microscopy until 70% of the cells were infected. The medium was replaced with DMEM without FBS, and cells were incubated for up to 2 days at 37°C in 5% CO_2_. Supernatant fluid was collected, centrifuged at 300 × *g* for 10 min at 4°C, aliquoted, and kept at −80°C until use. All recombinant viruses were sequenced at this stage using the same mNGS methods detailed above on 50 µl of supernatant fluid to confirm the correct CI backbone and HN mutation sequences before use in experiments.

### Viral propagation in human airway epithelia.

The HAE EpiAirway AIR-100 system (MatTek Corporation) consists of normal human-derived tracheo/bronchial epithelial cells that have been cultured to form a pseudostratified, highly differentiated mucociliary epithelium closely resembling that of epithelial tissue *in vivo*. Upon receipt from the manufacturer, human airway epithelium (HAE) cultures were transferred to six-well plates (containing 1 ml medium per well) with the apical surface remaining exposed to air and incubated at 37°C in 5% CO_2_. HAE cultures were infected with 5,000 PFU of recombinant clinical isolate 1 EGFP (5) (referred to as CI-1-EGFP-F K108) at the apical surface for 90 min at 37°C. The wild type (wt) CI-1-EGFP-F K108 or CI-1-EGFP-F K108 harboring single mutations in HN (i.e., P241L, R242K, H552Q, S554N, or L555F) was used. At 90 min, the medium containing the inoculum was removed, and cultures were placed at 37°C and fed each day with 1 ml medium via the basolateral surface. Viruses were harvested by adding 200 µl of 1× phosphate-buffered saline (PBS) with calcium and magnesium chloride (MatTek Corporation) per well to the HAE cultures’ apical surface and equilibrating for 30 min at 37°C. The suspension was then collected, and viral titers were determined as previously described (58). This viral collection was performed sequentially with the same wells of cells on each indicated day postinfection.

### Viral propagation in CV-1.

CV-1 cultures were seeded in 24-well plates. Seventy percent confluent cells were infected with 5,000 PFU of recombinant CI-1-EGFP-F K108 (wt or harboring single mutations in HN [i.e., P241L, R242K, H552Q, S554N, L555F]) at the apical surface for 90 min at 37°C. After 90 min, the medium containing the inoculum was replaced with 1 ml of DMEM supplemented with 5% FBS. The supernatant fluid was then collected, aliquoted, and replaced with the same volume of DMEM supplemented with 5% FBS, and viral titers were determined as previously described (58). This viral collection was performed sequentially with the same wells of cells on each indicated day postinfection.

## References

[1] Nair H, Nokes DJ, Gessner BD, Dherani M, Madhi SA, Singleton RJ, O’Brien KL, Roca A, Wright PF, Bruce N, Chandran A, Theodoratou E, Sutanto A, Sedyaningsih ER, Ngama M, Munywoki PK, Kartasasmita C, Simões EA, Rudan I, Weber MW, Campbell H 2010 Global burden of acute lower respiratory infections due to respiratory syncytial virus in young children: a systematic review and meta-analysis. Lancet 375:1545–1555. doi:10.1016/S0140-6736(10)60206-1. 20399493 PMC2864404

[2] Maziarz RT, Sridharan P, Slater S, Meyers G, Post M, Erdman DD, Peret TC, Taplitz RA 2010 Control of an outbreak of human parainfluenza virus 3 in hematopoietic stem cell transplant recipients. Biol Blood Marrow Transplant 16:192–198. doi:10.1016/j.bbmt.2009.09.014. 19781656 PMC7172256

[3] Liu WK, Liu Q, Chen DH, Liang HX, Chen XK, Huang WB, Qin S, Yang ZF, Zhou R 2013 Epidemiology and clinical presentation of the four human parainfluenza virus types. BMC Infect Dis 13:28. doi:10.1186/1471-2334-13-28. 23343342 PMC3560251

[4] Palermo LM, Uppal M, Skrabanek L, Zumbo P, Germer S, Toussaint NC, Rima BK, Huey D, Niewiesk S, Porotto M, Moscona A 2016 Features of circulating parainfluenza virus required for growth in human airway. mBio 7:e00235. doi:10.1128/mBio.00235-16. 26980833 PMC4807361

[5] Palmer SG, DeVito I, Jenkins SG, Niewiesk S, Porotto M, Moscona A 2014 Circulating clinical strains of human parainfluenza virus reveal viral entry requirements for in vivo infection. J Virol 88:13495–13502. doi:10.1128/JVI.01965-14. 25210187 PMC4249073

[6] Xu R, Palmer SG, Porotto M, Palermo LM, Niewiesk S, Wilson IA, Moscona A 2013 Interaction between the hemagglutinin-neuraminidase and fusion glycoproteins of human parainfluenza virus type III regulates viral growth in vivo. mBio 4:e00803-13. doi:10.1128/mBio.00803-13. 24149514 PMC3812707

[7] Palmer SG, Porotto M, Palermo LM, Cunha LF, Greengard O, Moscona A 2012 Adaptation of human parainfluenza virus to airway epithelium reveals fusion properties required for growth in host tissue. mBio 3:e00137-12. doi:10.1128/mBio.00137-12. 22669629 PMC3374391

[8] Palgen JL, Jurgens EM, Moscona A, Porotto M, Palermo LM 2015 Unity in diversity: shared mechanism of entry among paramyxoviruses. Prog Mol Biol Transl Sci 129:1–32. doi:10.1016/bs.pmbts.2014.10.001. 25595799 PMC4369139

[9] Porotto M, Murrell M, Greengard O, Moscona A 2003 Triggering of human parainfluenza virus 3 fusion protein (F) by the hemagglutinin-neuraminidase (HN) protein: an HN mutation diminishes the rate of F activation and fusion. J Virol 77:3647–3654. doi:10.1128/JVI.77.6.3647-3654.2003. 12610140 PMC149538

[10] Porotto M, Palmer SG, Palermo LM, Moscona A 2012 Mechanism of fusion triggering by human parainfluenza virus type III: communication between viral glycoproteins during entry. J Biol Chem 287:778–793. doi:10.1074/jbc.M111.298059. 22110138 PMC3249132

[11] Porotto M, Salah ZW, Gui L, DeVito I, Jurgens EM, Lu H, Yokoyama CC, Palermo LM, Lee KK, Moscona A 2012 Regulation of paramyxovirus fusion activation: the hemagglutinin-neuraminidase protein stabilizes the fusion protein in a pretriggered state. J Virol 86:12838–12848. doi:10.1128/JVI.01965-12. 22993149 PMC3497673

[12] Huberman K, Peluso RW, Moscona A 1995 The hemagglutinin-neuraminidase of human parainfluenza virus type 3: role of the neuraminidase in the viral life cycle. Virology 214:294–300. doi:10.1006/viro.1995.9925. 8525632

[13] Wu NC, Zost SJ, Thompson AJ, Oyen D, Nycholat CM, McBride R, Paulson JC, Hensley SE, Wilson IA 2017 A structural explanation for the low effectiveness of the seasonal influenza H3N2 vaccine. PLoS Pathog 13:e1006682. doi:10.1371/journal.ppat.1006682. 29059230 PMC5667890

[14] Parker L, Wharton SA, Martin SR, Cross K, Lin Y, Liu Y, Feizi T, Daniels RS, McCauley JW 2016 Effects of egg-adaptation on receptor-binding and antigenic properties of recent influenza A (H3N2) vaccine viruses. J Gen Virol 97:1333–1344. doi:10.1099/jgv.0.000457. 26974849 PMC5394856

[15] Kaul A, Woerz I, Meuleman P, Leroux-Roels G, Bartenschlager R 2007 Cell culture adaptation of hepatitis C virus and in vivo viability of an adapted variant. J Virol 81:13168–13179. doi:10.1128/JVI.01362-07. 17881454 PMC2169131

[16] Dargan DJ, Douglas E, Cunningham C, Jamieson F, Stanton RJ, Baluchova K, McSharry BP, Tomasec P, Emery VC, Percivalle E, Sarasini A, Gerna G, Wilkinson GW, Davison AJ 2010 Sequential mutations associated with adaptation of human cytomegalovirus to growth in cell culture. J Gen Virol 91:1535–1546. doi:10.1099/vir.0.018994-0. 20479471 PMC3052722

[17] Guarnaccia T, Carolan LA, Maurer-Stroh S, Lee RT, Job E, Reading PC, Petrie S, McCaw JM, McVernon J, Hurt AC, Kelso A, Mosse J, Barr IG, Laurie KL 2013 Antigenic drift of the pandemic 2009 A(H1N1) influenza virus in a ferret model. PLoS Pathog 9:e1003354. doi:10.1371/journal.ppat.1003354. 23671418 PMC3649996

[18] Andersen KG, Shapiro BJ, Matranga CB, Sealfon R, Lin AE, Moses LM, Folarin OA, Goba A, Odia I, Ehiane PE, Momoh M, England EM, Winnicki S, Branco LM, Gire SK, Phelan E, Tariyal R, Tewhey R, Omoniwa O, Fullah M, Fonnie R, Fonnie M, Kanneh L, Jalloh S, Gbakie M, Saffa S, Karbo K, Gladden AD, Qu J, Stremlau M, Nekoui M, Finucane HK, Tabrizi S, Vitti JJ, Birren B, Fitzgerald M, McCowan C, Ireland A, Berlin AM, Bochicchio J, Tazon-Vega B, Lennon NJ, Ryan EM, Bjornson Z, Milner DA Jr, Lukens AK, Broodie N, Rowland M, Heinrich M, Akdag M, Schieffelin JS, et al. 2015 Clinical sequencing uncovers origins and evolution of Lassa virus. Cell 162:738–750. doi:10.1016/j.cell.2015.07.020. 26276630 PMC4537774

[19] Bateman AC, Greninger AL, Atienza EE, Limaye AP, Jerome KR, Cook L 2017 Quantification of BK virus standards by quantitative real-time PCR and droplet digital PCR is confounded by multiple virus populations in the WHO BKV international standard. Clin Chem 63:761–769. doi:10.1373/clinchem.2016.265512. 28100494

[20] Greninger AL, Bateman AC, Atienza EE, Wendt S, Makhsous N, Jerome KR, Cook L 2017 Copy number heterogeneity of JC virus standards. J Clin Microbiol 55:824–831. doi:10.1128/JCM.02337-16. 27974546 PMC5328450

[21] Xue KS, Greninger AL, Pérez-Osorio A, Bloom JD 2018 Cooperating H3N2 influenza virus variants are not detectable in primary clinical samples. mSphere 3:e00552-17. doi:10.1128/mSphereDirect.00552-17. 29299533 PMC5750391

[22] Ogimi C, Greninger AL, Waghmare AA, Kuypers JM, Shean RC, Xie H, Leisenring WM, Stevens-Ayers TL, Jerome KR, Englund JA, Boeckh M 2017 Prolonged shedding of human coronavirus in hematopoietic cell transplant recipients: risk factors and viral genome evolution. J Infect Dis 216:203–209. doi:10.1093/infdis/jix264. 28838146 PMC5853311

[23] Tang JW, Lam TT, Zaraket H, Lipkin WI, Drews SJ, Hatchette TF, Heraud JM, Koopmans MP, INSPIRE Investigators 2017 Global epidemiology of non-influenza RNA respiratory viruses: data gaps and a growing need for surveillance. Lancet Infect Dis 17:e320–e326. doi:10.1016/S1473-3099(17)30238-4. 28457597 PMC7164797

[24] Greninger AL 2018 A decade of RNA virus metagenomics is (not) enough. Virus Res 244:218–229. doi:10.1016/j.virusres.2017.10.014. 29055712 PMC7114529

[25] McCrone JT, Lauring AS 2016 Measurements of intrahost viral diversity are extremely sensitive to systematic errors in variant calling. J Virol 90:6884–6895. doi:10.1128/JVI.00667-16. 27194763 PMC4944299

[26] Porotto M, Fornabaio M, Kellogg GE, Moscona A 2007 A second receptor binding site on human parainfluenza virus type 3 hemagglutinin-neuraminidase contributes to activation of the fusion mechanism. J Virol 81:3216–3228. doi:10.1128/JVI.02617-06. 17229690 PMC1866072

[27] Moscona A, Peluso RW 1993 Relative affinity of the human parainfluenza virus type 3 hemagglutinin-neuraminidase for sialic acid correlates with virus-induced fusion activity. J Virol 67:6463–6468. 8411349 10.1128/jvi.67.11.6463-6468.1993 PMC238082

[28] Mizuta K, Tsukagoshi H, Ikeda T, Aoki Y, Abiko C, Itagaki T, Nagano M, Noda M, Kimura H 2014 Molecular evolution of the haemagglutinin-neuraminidase gene in human parainfluenza virus type 3 isolates from children with acute respiratory illness in Yamagata Prefecture, Japan. J Med Microbiol 63:570–577. doi:10.1099/jmm.0.068189-0. 24464692

[29] Martin DP, Murrell B, Golden M, Khoosal A, Muhire B 2015 RDP4: detection and analysis of recombination patterns in virus genomes. Virus Evol 1:vev003. doi:10.1093/ve/vev003. 27774277 PMC5014473

[30] Han GZ, Worobey M 2011 Homologous recombination in negative sense RNA viruses. Viruses 3:1358–1373. doi:10.3390/v3081358. 21994784 PMC3185808

[31] Pomeroy LW, Bjørnstad ON, Holmes EC 2008 The evolutionary and epidemiological dynamics of the Paramyxoviridae. J Mol Evol 66:98–106. doi:10.1007/s00239-007-9040-x. 18217182 PMC3334863

[32] Roth JP, Li JK, Smee DF, Morrey JD, Barnard DL 2009 A recombinant, infectious human parainfluenza virus type 3 expressing the enhanced green fluorescent protein for use in high-throughput antiviral assays. Antiviral Res 82:12–21. doi:10.1016/j.antiviral.2009.01.001. 19189850 PMC2701465

[33] van Wyke Coelingh KL, Winter CC, Murphy BR 1988 Nucleotide and deduced amino acid sequence of hemagglutinin-neuraminidase genes of human type 3 parainfluenza viruses isolated from 1957 to 1983. Virology 162:137–143. doi:10.1016/0042-6822(88)90402-3. 2827373

[34] Day ND, Branigan PJ, Liu C, Gutshall LL, Luo J, Melero JA, Sarisky RT, Del Vecchio AM 2006 Contribution of cysteine residues in the extracellular domain of the F protein of human respiratory syncytial virus to its function. Virol J 3:34. doi:10.1186/1743-422X-3-34. 16723026 PMC1540417

[35] Ohsawa K, Yamada A, Takeuchi K, Watanabe Y, Miyata H, Sato H 1998 Genetic characterization of parainfluenza virus 3 derived from guinea pigs. J Vet Med Sci 60:919–922. doi:10.1292/jvms.60.919. 9764404

[36] Tappert MM, Smith DF, Air GM 2011 Fixation of oligosaccharides to a surface may increase the susceptibility to human parainfluenza virus 1, 2, or 3 hemagglutinin-neuraminidase. J Virol 85:12146–12159. doi:10.1128/JVI.05537-11. 21917945 PMC3209406

[37] Du RP, Jackson GE, Wyde PR, Yan WY, Wang Q, Gisonni L, Sanhueza SE, Klein MH, Ewasyshyn ME 1994 A prototype recombinant vaccine against respiratory syncytial virus and parainfluenza virus type 3. Biotechnology 12:813–818. doi:10.1038/nbt0894-813. 7765021

[38] Berrueco R, Antón A, Rives S, Català A, Toll T, Ruiz A, Camós M, Torrebadell M, Estella J, Muñoz-Almagro C 2013 Multiplex real-time PCR for prompt diagnosis of an outbreak of human parainfluenza 3 virus in children with acute leukemia. Infection 41:1171–1175. doi:10.1007/s15010-013-0498-8. 23821486 PMC7100800

[39] Mahon PJ, Mirza AM, Musich TA, Iorio RM 2008 Engineered intermonomeric disulfide bonds in the globular domain of Newcastle disease virus hemagglutinin-neuraminidase protein: implications for the mechanism of fusion promotion. J Virol 82:10386–10396. doi:10.1128/JVI.00581-08. 18753211 PMC2573173

[40] Palermo LM, Porotto M, Greengard O, Moscona A 2007 Fusion promotion by a paramyxovirus hemagglutinin-neuraminidase protein: pH modulation of receptor avidity of binding sites I and II. J Virol 81:9152–9161. doi:10.1128/JVI.00888-07. 17567695 PMC1951465

[41] Greengard O, Poltoratskaia N, Leikina E, Zimmerberg J, Moscona A 2000 The anti-influenza virus agent 4-GU-DANA (zanamivir) inhibits cell fusion mediated by human parainfluenza virus and influenza virus HA. J Virol 74:11108–11114. doi:10.1128/JVI.74.23.11108-11114.2000. 11070006 PMC113191

[42] Porotto M, Murrell M, Greengard O, Lawrence MC, McKimm-Breschkin JL, Moscona A 2004 Inhibition of parainfluenza virus type 3 and Newcastle disease virus hemagglutinin-neuraminidase receptor binding: effect of receptor avidity and steric hindrance at the inhibitor binding sites. J Virol 78:13911–13919. doi:10.1128/JVI.78.24.13911-13919.2004. 15564499 PMC533954

[43] Porotto M, Fornabaio M, Greengard O, Murrell MT, Kellogg GE, Moscona A 2006 Paramyxovirus receptor-binding molecules: engagement of one site on the hemagglutinin-neuraminidase protein modulates activity at the second site. J Virol 80:1204–1213. doi:10.1128/JVI.80.3.1204-1213.2006. 16414997 PMC1346948

[44] Zaitsev V, von Itzstein M, Groves D, Kiefel M, Takimoto T, Portner A, Taylor G 2004 Second sialic acid binding site in Newcastle disease virus hemagglutinin-neuraminidase: implications for fusion. J Virol 78:3733–3741. doi:10.1128/JVI.78.7.3733-3741.2004. 15016893 PMC371092

[45] Benson DA, Cavanaugh M, Clark K, Karsch-Mizrachi I, Ostell J, Pruitt KD, Sayers EW 2018 GenBank. Nucleic Acids Res 46:D41–D47. doi:10.1093/nar/gkx1094. 29140468 PMC5753231

[46] Wheeler DL, Barrett T, Benson DA, Bryant SH, Canese K, Chetvernin V, Church DM, Dicuccio M, Edgar R, Federhen S, Feolo M, Geer LY, Helmberg W, Kapustin Y, Khovayko O, Landsman D, Lipman DJ, Madden TL, Maglott DR, Miller V, Ostell J, Pruitt KD, Schuler GD, Shumway M, Sequeira E, Sherry ST, Sirotkin K, Souvorov A, Starchenko G, Tatusov RL, Tatusova TA, Wagner L, Yaschenko E 2008 Database resources of the National Center for Biotechnology Information. Nucleic Acids Res 36:D13–D21. doi:10.1093/nar/gkm1000. 18045790 PMC2238880

[47] Greninger AL, Messacar K, Dunnebacke T, Naccache SN, Federman S, Bouquet J, Mirsky D, Nomura Y, Yagi S, Glaser C, Vollmer M, Press CA, Kleinschmidt-DeMasters BK, Dominguez SR, Chiu CY 2015 Clinical metagenomic identification of Balamuthia mandrillaris encephalitis and assembly of the draft genome: the continuing case for reference genome sequencing. Genome Med 7:113. doi:10.1186/s13073-015-0235-2. 26620704 PMC4665321

[48] Greninger AL, Naccache SN, Federman S, Yu G, Mbala P, Bres V, Stryke D, Bouquet J, Somasekar S, Linnen JM, Dodd R, Mulembakani P, Schneider BS, Muyembe-Tamfum JJ, Stramer SL, Chiu CY 2015 Rapid metagenomic identification of viral pathogens in clinical samples by real-time nanopore sequencing analysis. Genome Med 7:99. doi:10.1186/s13073-015-0220-9. 26416663 PMC4587849

[49] Greninger AL, Zerr DM, Qin X, Adler AL, Sampoleo R, Kuypers JM, Englund JA, Jerome KR 2017 Rapid metagenomic next-generation sequencing during an investigation of hospital-acquired human parainfluenza virus 3 infections. J Clin Microbiol 55:177–182. doi:10.1128/JCM.01881-16. 27795347 PMC5228228

[50] Greninger AL, Chen EC, Sittler T, Scheinerman A, Roubinian N, Yu G, Kim E, Pillai DR, Guyard C, Mazzulli T, Isa P, Arias CF, Hackett J, Schochetman G, Miller S, Tang P, Chiu CY 2010 A metagenomic analysis of pandemic influenza A (2009 H1N1) infection in patients from North America. PLoS One 5:e13381. doi:10.1371/journal.pone.0013381. 20976137 PMC2956640

[51] Greninger AL, Waghmare A, Adler A, Qin X, Crowley JL, Englund JA, Kuypers JM, Jerome KR, Zerr DM 2017 Rule-out outbreak: 24-hour metagenomic next-generation sequencing for characterizing respiratory virus source for infection prevention. J Pediatr Infect Dis Soc 6:168–172. doi:10.1093/jpids/pix019. PMC5907853 28379561

[52] Greninger AL, Pepper G, Shean RC, Cent A, Palileo I, Kuypers JM, Schiffer JT, Jerome KR 2017 Myeloablation-associated deletion of ORF4 in a human coronavirus 229E infection. NPJ Genom Med 2:30. doi:10.1038/s41525-017-0033-4. 29263840 PMC5677986

[53] Katoh K, Misawa K, Kuma K, Miyata T 2002 MAFFT: a novel method for rapid multiple sequence alignment based on fast Fourier transform. Nucleic Acids Res 30:3059–3066. doi:10.1093/nar/gkf436. 12136088 PMC135756

[54] Drummond AJ, Suchard MA, Xie D, Rambaut A 2012 Bayesian phylogenetics with BEAUti and the BEAST 1.7. Mol Biol Evol 29:1969–1973. doi:10.1093/molbev/mss075. 22367748 PMC3408070

[55] Rambaut A, Xie D, Drummond AJ 2014 Tracer v1.6. http://tree.bio.ed.ac.uk/software/tracer/.

[56] Horga MA, Gusella GL, Greengard O, Poltoratskaia N, Porotto M, Moscona A 2000 Mechanism of interference mediated by human parainfluenza virus type 3 infection. J Virol 74:11792–11799. doi:10.1128/JVI.74.24.11792-11799.2000. 11090179 PMC112462

[57] Zhang L, Bukreyev A, Thompson CI, Watson B, Peeples ME, Collins PL, Pickles RJ 2005 Infection of ciliated cells by human parainfluenza virus type 3 in an in vitro model of human airway epithelium. J Virol 79:1113–1124. doi:10.1128/JVI.79.2.1113-1124.2005. 15613339 PMC538579

[58] Palermo LM, Porotto M, Yokoyama CC, Palmer SG, Mungall BA, Greengard O, Niewiesk S, Moscona A 2009 Human parainfluenza virus infection of the airway epithelium: the viral hemagglutinin-neuraminidase regulates fusion protein activation and modulates infectivity. J Virol 83:6900–6908. doi:10.1128/JVI.00475-09. 19386708 PMC2698534

