# Supplementary material for: Viral Entry Properties Required for Fitness in Humans Are Lost through Rapid Genomic Change during Viral Isolation
Source: mBio. 2018 Jul 3;9(4):e00898-18. doi: 10.1128/mBio.00898-18 (PMC6030562; doi:10.1128/mBio.00898-18)
Supplement: TABLE S1 [file mbo003183958st1.pdf]

Table S1. Sample metadata

Accession	Strain	Collection Date	Background	Collection Type
KY369862	SC129	2016	Clinical Sample	Nasal Swab
KY369863	SC161	2016	Clinical Sample	Nasal Swab
KY369864	SC291	2016	Clinical Sample	Nasal Swab
KY369865	SC789	2016	Clinical Sample	Nasal Swab
KY369866	SC876	2016	Clinical Sample	Nasal Swab
KY369867	SC1216	2016	Clinical Sample	Nasal Swab
KY369868	SC139	2016	Clinical Sample	Nasal Swab
KY369869	SC271	2016	Clinical Sample	Nasal Swab
KY369870	SC332	2016	Clinical Sample	Nasal Swab
KY369871	SC717	2016	Clinical Sample	Nasal Swab
KY369872	SC9791	2016	Clinical Sample	Nasal Swab
KY369876	SC113	2016	Clinical Sample	Nasal Swab
KY629772	16-J1	2016	Culture Isolate	BAL
KY629774	16-J5	2016	Culture Isolate	BAL
KY674922	16G3	2016	Culture Isolate	Nasal Swab
KY674923	9T9	2009	Culture Isolate	BAL
KY674924	9T7	2009	Culture Isolate	Nasal Wash
KY674925	9T3	2009	Culture Isolate	BAL
KY674926	9T2	2009	Culture Isolate	Nasal Swab
KY674927	9T8	2009	Culture Isolate	Nasal Wash
KY674928	9S5	2009	Culture Isolate	Nasal Wash
KY674929	9R4	2009	Culture Isolate	Nasal Wash
KY674930	9R3	2009	Culture Isolate	Sputum
KY674931	9Q10	2009	Culture Isolate	BAL
KY674932	9Q6	2009	Culture Isolate	Nasal Wash
KY674933	9Q4	2009	Culture Isolate	Nasal Wash
KY674934	9Q3	2009	Culture Isolate	Nasal Wash
KY674935	9Q2	2009	Culture Isolate	BAL
KY674936	9Q1	2009	Culture Isolate	BAL
KY674937	9P8	2009	Culture Isolate	Nasal Wash
KY674938	9P6	2009	Culture Isolate	Nasal Wash
KY674939	9P5	2009	Culture Isolate	Nasal Wash
KY674963	12M7	2012	Culture Isolate	BAL
KY674964	12N3	2012	Culture Isolate	Nasal Swab
KY674975	16C7	2016	Culture Isolate	Nasal Swab
KY674977	16D1	2016	Culture Isolate	Nasal Swab
KY674978	16D10	2016	Culture Isolate	BAL
KY674979	16E1	2016	Culture Isolate	Nasal Swab

KY674981	16E8	2016	Culture Isolate	Nasal Swab
KY674982	16F7	2016	Culture Isolate	CAP Proficiency Testing
KY684744	9E7	2009	Culture Isolate	Nasal Wash
KY684745	9C2	2009	Culture Isolate	unknown
KY684746	9D2	2009	Culture Isolate	Nasal Wash
KY684747	9F7	2009	Culture Isolate	Nasal Wash
KY684748	9F8	2009	Culture Isolate	Nasal Wash
KY684749	9G3	2009	Culture Isolate	Nasal Wash
KY684750	9I8	2009	Culture Isolate	Nasal Swab
KY684751	9I9	2009	Culture Isolate	Nasal
KY684752	9K9	2009	Culture Isolate	Nasal Wash
KY684753	9L10	2009	Culture Isolate	Nasal Wash
KY684754	9M6	2009	Culture Isolate	Nasal Wash
KY684755	SC2155	2016	Clinical Sample	Nasal Swab
KY684756	SC2171	2016	Clinical Sample	Nasal Swab
KY684761	9M7	2009	Culture Isolate	unk
KY973568	10E1	2010	Culture Isolate	BAL
KY973569	SC3238	2015	Clinical Sample	Nasal Swab
KY973570	SC2565	2015	Clinical Sample	Nasal Wash
KY973571	SC2337	2015	Clinical Sample	Nasal Swab
KY973572	10S2	2010	Culture Isolate	Nasal Wash
KY973573	10Q8	2010	Culture Isolate	BAL
KY973574	10Q7	2010	Culture Isolate	BAL
KY973575	10P9	2010	Culture Isolate	Nasal Swab
KY973576	10P1	2010	Culture Isolate	Nasal Wash
KY973577	10N7	2010	Culture Isolate	Nasal Wash
KY973578	10N6	2010	Culture Isolate	Nasal Swab
KY973579	10M9	2010	Culture Isolate	Nasal Wash
KY973580	10L8	2010	Culture Isolate	BAL
KY973581	10L6	2010	Culture Isolate	Nasal Wash
KY973582	10L5	2010	Culture Isolate	Nasal Wash
KY973583	10L3	2010	Culture Isolate	Nasal Wash
KY973584	10J10	2010	Culture Isolate	Nasal Wash
KY973585	10J5	2010	Culture Isolate	Nasal Wash
KY973586	10J4	2010	Culture Isolate	Nasal Wash
KY973587	10J3	2010	Culture Isolate	Nasal Wash
KY973588	10I8	2010	Culture Isolate	Nasal Wash

KY973589	10I6	2010	Culture Isolate	Throat Swab
KY973590	10I5	2010	Culture Isolate	Nasal Wash
KY973591	10I4	2010	Culture Isolate	Nasal Swab
KY973592	10H8	2010	Culture Isolate	unknown
KY973593	10G10	2010	Culture Isolate	BAL
KY973594	10F5	2010	Culture Isolate	Nasal Wash
KY973595	10F1	2010	Culture Isolate	Nasal Wash
KY973596	10E9	2010	Culture Isolate	BAL
KY973597	10E6	2010	Culture Isolate	Nasal Wash
KY973598	10E5	2010	Culture Isolate	Nasal Wash
KY973599	10E4	2010	Culture Isolate	Nasal Wash
KY973600	10E2	2010	Culture Isolate	BAL
KY986641	10J1	2010	Culture Isolate	Nasal Wash
KY986642	10K3	2010	Culture Isolate	Nasal Wash
KY986643	10L2	2010	Culture Isolate	Nasal Wash
MF166751	SC3182	2015	Clinical Sample	BAL
MF166752	SC3331	2015	Clinical Sample	Nasal Swab
MF554716	2D5	2009	Culture Isolate	BAL
MF554717	2A8	2009	Culture Isolate	BAL
MF554718	2T2	2009	Culture Isolate	Nasal Wash
MF554719	2T1	2009	Culture Isolate	Nasal Wash
MF554720	2P2	2009	Culture Isolate	Nasal Wash
MF554721	2P1	2009	Culture Isolate	Nasal Wash
MF554722	2O10	2009	Culture Isolate	Nasal Wash
MF554723	2O8	2009	Culture Isolate	Nasal Wash
MF554724	2O7	2009	Culture Isolate	Nasal Wash
MF554725	2F10	2009	Culture Isolate	Nasal Wash
MF554726	2F9	2009	Culture Isolate	Nasal Wash
MF554727	2F8	2009	Culture Isolate	Nasal Wash
MF554728	2F6	2009	Culture Isolate	Nasal Wash
MF554729	2D3	2009	Culture Isolate	Nasal Wash
MF554730	2C8	2009	Culture Isolate	Nasal Wash
MF554731	2C5	2009	Culture Isolate	Nasal Wash
MF554732	2B7	2009	Culture Isolate	Nasal Wash
MF554733	2B6	2009	Culture Isolate	BAL
MF554734	2B5	2009	Culture Isolate	Nasal Swab
MF554735	2B1	2009	Culture Isolate	Nasal Wash
MF554736	2E6	2009	Culture Isolate	Nasal Wash
MF795094	SC1201	2015	Clinical Sample	Nasal Wash
MF795095	SC1784	2015	Clinical Sample	BAL

MF795096	SC2711	2015	Clinical Sample	Nasal Swab
MF795097	SC2288	2015	Clinical Sample	Nasal Swab
MF973163	7N2	2011	Culture Isolate	Nasal Wash
